# A Clinical Genetics-Driven Dual Diagnosis of Prader–Willi Syndrome Due to Mosaic Maternal UPD(15) and *NOTCH3*-Related CADASIL

**DOI:** 10.3390/genes17080937

**Published:** 2026-08-11

**Authors:** Francesco Maria Bogliardi, Pino D’Ambrosio, Giorgia Quattromini, Giordana Di Mario, Maria Grazia Pomponi, Luca Miele, Edoardo Vergani, Giuseppe Zampino, Antonio Liguori, Marcella Zollino, Antonino Crinò

**Affiliations:** 1 Section of Genomic Medicine, Department of Life Sciences and Public Health, Università Cattolica del Sacro Cuore, 00168 Rome, Italy; 2 Medical Genetics Unit, Fondazione Policlinico Universitario “A. Gemelli” IRCCS, 00168 Rome, Italy; 3 CEMAD Digestive Diseases Center, Fondazione Policlinico Universitario “A. Gemelli” IRCCS, 00168 Rome, Italy; 4 Complex Operative Unit of Internal Medicine, Endocrinology and Diabetology, Fondazione Policlinico Universitario “A. Gemelli” IRCCS, 00168 Rome, Italy; 5 Centre for Rare Diseases and Congenital Defects, Fondazione Policlinico Universitario “A. Gemelli” IRCCS, 00168 Rome, Italy

**Keywords:** Prader–Willi syndrome, mosaicism, uniparental disomy, SNP-array, CADASIL, *NOTCH3*, focal segmental glomerulosclerosis

## Abstract

Maternal uniparental disomy of chromosome 15 [UPD(15)mat] and imprinting defects account for about 30% of cases of Prader–Willi syndrome (PWS). Mosaic UPD(15)mat is rare and may escape routine testing. We describe a 45-year-old male patient in whom persistent clinical suspicion of PWS was not genetically confirmed by repeated methylation-based analyses. Clinical manifestations included neonatal hypotonia with low birth weight, early hyperphagia, severe obesity, short stature, growth hormone deficiency, type 2 diabetes mellitus, dyslipidemia, and MASLD/MASH with compensated cirrhosis. He presented with very mild neurodevelopmental impairment. Following the detection of proteinuria and microalbuminuria from age 22 years, focal segmental glomerulosclerosis was diagnosed upon renal biopsy. A family history of cerebrovascular events was recorded. Combined SNP-array and MS-MLPA analyses across tissues established a diagnosis of PWS due to mosaic UPD(15)mat. The mosaic fraction, estimated by SNP-array, was approximately 10% in peripheral blood and 40% in buccal cells; MS-MLPA detected abnormal methylation only in buccal cells, explaining the previous negative blood-based results. Exome sequencing identified the paternally inherited pathogenic *NOTCH3* variant NM_000435.2:c.3016C>T, p.(Arg1006Cys). Subsequent brain MRI showed chronic vascular-type leukoencephalopathy consistent with CADASIL, despite the absence of overt ischemic events in the proband. Collectively, these investigations established a dual molecular diagnosis of PWS due to mosaic UPD(15)mat and *NOTCH3*-related CADASIL. This report highlights the pivotal role of clinical genetics in assessing the precise diagnosis in rare diseases. With respect to PWS, it demonstrates that mosaicism can lead to a missed diagnosis when the genetic investigation is limited to peripheral blood. In addition, following the diagnosis of CADASIL, and based on the available evidence linking NOTCH3 to renal physiology and disease, we discuss whether *NOTCH3*-related renal microangiopathy may have contributed to the renal phenotype. However, given the patient’s multiple renal risk factors, FSGS was considered most likely multifactorial, and a causal association with CADASIL cannot be established from this single case.

## 1. Introduction

Prader–Willi syndrome (PWS) is a complex imprinting disorder characterized by severe neonatal hypotonia and feeding difficulties, followed by developmental delays of variable degrees, hyperphagia, obesity, short stature, hypogonadism, growth hormone deficiency, and a broad range of endocrine, metabolic, and behavioral complications [1,2]. This disorder results from the loss of expression of paternally expressed genes in the 15q11.2-q13 PWS critical region. The underlying mechanisms include deletion of the paternal allele, maternal uniparental disomy of chromosome 15 [UPD(15)mat], and epigenetic defects [1,3].

Current diagnostic algorithms rely on DNA methylation analysis to identify maternal-only imprinting at 15q11.2-q13. However, methylation testing alone does not fully define the molecular mechanism, and combined approaches including oligo-SNP array and methylation-specific multiplex ligation-dependent probe amplification (MS-MLPA) are recommended to characterize deletions, isodisomy, segmental isodisomy, and selected epigenetic defects [1,4]. Mosaicism adds a further diagnostic challenge. In mosaic UPD(15)mat, the proportion of abnormal cells may be low in blood. Thus, in the presence of a likely clinical diagnosis of PWS, normal genetic results on peripheral blood cells cannot be considered exhaustive [5,6,7,8,9,10,11,12].

Cerebral autosomal dominant arteriopathy with subcortical infarcts and leukoencephalopathy (CADASIL) is the most common monogenic cerebral small-vessel disease. It is caused by heterozygous pathogenic variants in *NOTCH3*, most commonly including missense variants that alter the number of cysteine residues in epidermal growth factor-like repeats of the extracellular domain [13,14]. This disorder is characterized by recurrent lacunar ischemic events, migraine with aura, cognitive decline, psychiatric manifestations, and progressive white matter hyperintensities on brain MRI [13,15].

We report the case of an adult male patient in whom a clinical genetics-driven procedure led to a dual diagnosis of PWS due to mosaic UPD(15)mat and *NOTCH3*-related CADASIL. To the best of our knowledge, this is the first reported coexistence of PWS and molecularly confirmed *NOTCH3*-related CADASIL. We also discuss a possible, but unproven, contribution of *NOTCH3*-related arteriopathy to focal segmental glomerulosclerosis (FSGS) in this patient, based on published data on renal involvement in CADASIL and experimental evidence implicating Notch3 signaling in glomerular injury.

## 2. Detailed Case Description

### 2.1. Clinical History and Endocrine-Metabolic Phenotype

The proband is a 45-year-old male, the second child of non-consanguineous parents. His family history was unremarkable for neurodevelopmental disorders and congenital anomalies. His family history was notable for transient ischemic attacks and strokes affecting several relatives on the paternal side.

Reduced fetal movements and poor fetal growth were reported in our proband. According to the maternal history, no exposure to known teratogenic medications, alcohol, tobacco, illicit drugs, or ionizing radiation and no relevant maternal infections were reported during pregnancy. He was born at term after spontaneous dystocic delivery, with a birth weight of 2450 g (z = −2.0). The neonatal period was complicated by perinatal respiratory distress, poor crying, marked hypotonia, poor sucking, and a two-week stay in an incubator. Feeding difficulties persisted during the first year of life.

Motor milestones were delayed. He walked independently at approximately 28–30 months and spoke his first words around three years of age. He underwent physical therapy from 18 months, with positive results. Formal cognitive testing was not performed. However, his developmental delay appears to be mild/borderline: he completed lower secondary school with educational support and is currently working independently as a janitor in a public administrative office. He is able to go out alone and use public transport, but he has limited autonomy in financial management.

He experienced hyperphagia from one year of age onwards. Hyperphagia was particularly severe during childhood, with insatiable hunger reported from approximately six to seven years of age. Progressive weight gain occurred from the first years of life. Upon clinical evaluation at age 45.8 years, his height was 154 cm (z = −3.16) and his weight was 97.5 kg (z = +1.73), resulting in a body mass index (BMI) of 41.1 kg/m^2^ (z = +2.46) and a head circumference of 58 cm (z = +2.0).

A constellation of minor physical anomalies was detected, including elongated face, frontal androgenetic alopecia, medially thick and laterally sparse eyebrows with synophrys, narrowed palpebral fissures, mild palpebral ptosis, a thin nose with a prominent bridge and elongated tip, prominent nasolabial folds, thin lips, a protruding chin, large anteverted ears, small hands and feet, and bilateral syndactyly of the second and third toes.

Endocrine anomalies included unilateral cryptorchidism, which was surgically corrected at four years of age, delayed puberty, with onset at 16 years, growth hormone deficiency, and pituitary hypoplasia, as detected by brain MRI at age 18. Discontinuous growth hormone therapy was administered from 12 to 17 years of age. Additional clinical manifestations included ophthalmological anomalies—in particular, myopic astigmatism and strabismus—and adolescence-onset scoliosis and kyphosis.

The patient experienced a long-lasting history of severe metabolic derangement. Impaired glucose tolerance with marked hyperinsulinemia was diagnosed at 13.8 years of age, with a fasting insulin concentration as high as 298 µU/mL (reference range 2–25 µU/mL). He developed type 2 diabetes mellitus at approximately 20 years of age, with HOMA-IR (Homeostatic Model Assessment of Insulin Resistance) values around 6–8 (a score higher than 3, indicating severe insulin resistance), considerably high insulin requirement (up to 190 units/day), and HbA1c (Hemoglobin A1c) values up to 9.7–12% (reference range below 5.75%). Additional comorbidities included dyslipidemia from childhood onwards, early arterial hypertension (adolescence), hyperuricemia, elevated homocysteine, and low testosterone. At age 45, HbA1c was 9.7%, fasting glucose was 160 mg/dL (reference range 60–100 mg/dL), HDL cholesterol was 37 mg/dL (optimal value above 60 mg/dL), and testosterone was 1.83 ng/mL (reference range 3 to 10 ng/mL).

He also presented with hepatic impairment, consisting of metabolic dysfunction-associated steatotic liver disease (MASLD), with biopsy-proven metabolic dysfunction-associated steatohepatitis (MASH; NAS 4/8, portal and perisinusoidal fibrosis), evolving toward compensated cirrhosis. Renal dysfunction was first diagnosed at 22 years of age, consisting of proteinuria and microalbuminuria. On renal biopsy performed at age 28 years, focal segmental glomerulosclerosis (FSGS), arteriolar intimal hyalinosis, areas of tubular atrophy associated with interstitial fibrosis, and mild lymphocytic infiltrate were detected, associated with only a trace of IgM/C3 deposits as observed by direct immunofluorescence. The patient had post-void residual urine and recurrent urinary tract infections and underwent intermittent bladder catheterization.

The anti-obesity and antidiabetic treatments included metformin, acarbose, insulin, liraglutide, semaglutide, and high-dose semaglutide. High-dose semaglutide (2.4 mg weekly) was not associated with q meaningful reduction in appetite, weight, or HbA1c. Tirzepatide was started in late 2025 and gradually increased in dosage; after approximately six months of follow-up, the patient showed appetite reduction, improved glycemic values, and weight reduction to approximately 93 kg (BMI = 39.2, z = +2.28), with diarrhea and a tendency towards hypoglycemia. Behavioral features were substantially unchanged. The main clinical and diagnostic milestones are summarized in Table 1.

### 2.2. Genetic Testing Methods and Results

The patient underwent several genetic investigations over time. Conventional chromosome analysis was performed at age 2.1 years, with normal results. Because of severe neonatal hypotonia, poor sucking, and early feeding difficulties, a clinical hypothesis of PWS was first raised at 18 months, but no molecular test capable of detecting all the causative mechanisms of this condition was available at that time. Methylation-sensitive Southern blotting for PWS on DNA from peripheral blood cells, performed at age 14.7 years, gave normal results. A gene panel analysis was subsequently performed. Library preparation was carried out using the NimbleGen SeqCap Target Enrichment kit (Roche NimbleGen, Madison, WI, USA), and sequencing was undertaken on Illumina platforms. Bioinformatic analyses included Burrows–Wheeler alignment to the GRCh37 reference genome, variant filtering, and prioritization using the TGex—Knowledge Driven NGS Analysis software version 3.4.1 (LifeMap Sciences, Alameda, CA, USA). The NM_170784.3:c.724G>T, p.(Ala242Ser) variant of uncertain significance in the *MKKS/BBS6* gene was detected in the heterozygous state. Biallelic variants in this gene cause autosomal recessive Bardet–Biedl syndrome type 6 and McKusick–Kaufman syndrome (OMIM *604896), both presenting with a markedly different clinical phenotype. No second variant was identified, and the presence of a cryptic pathogenic allele was considered unlikely on clinical grounds.

Additional DNA methylation analyses for PWS were performed on DNA from peripheral blood cells on three further occasions, all with normal results: methylation analysis at the D15S63/*SNRPN* loci at age 19.4 years, MLPA at age 30.5 years, and MS-MLPA at age 40 years. Despite these negative results, the clinical suspicion of PWS remained strong because of the combination of neonatal hypotonia with poor sucking, early-onset hyperphagia and severe obesity, short stature, growth hormone deficiency, hypogonadism, and relatively mild neurodevelopmental involvement. The genetic diagnosis was achieved at age 40 years, by combining SNP-array and MS-MLPA on different tissues. SNP-array on peripheral blood was consistent with a mosaic maternal isoUPD(15), with an estimated mosaicism level of approximately 10%. An analysis of buccal smear cells showed a higher mosaicism level, estimated at approximately 40%. These mosaicism levels were estimated by the diagnostic laboratory from the SNP-array profiles and should therefore be regarded as approximate estimates rather than absolute cell fractions. For SNP-array, the Illumina CytoSNP-850K kit was used and data analysis was performed with BlueFuse Multi Software Edition 4.4 (Illumina, San Diego, CA, USA). MS-MLPA of the 15q11.2-q13 region (SALSA MS-MLPA Probemix ME028-C1, MRC Holland, Amsterdam, The Netherlands) was able to detect hypermethylation in buccal cells but not in peripheral blood. A final diagnosis of PWS due to mosaic UPD(15)mat was made.

Of note, the metabolic impairment, including the severe hepatic dysfunction, was more severe than expected for this level of mosaicism, although a higher degree of mosaicism in the hypothalamus cannot be excluded. Moreover, the clinical presentation was more complex than in typical PWS. This consideration, along with the family history of vascular events, led us to hypothesize a comorbidity with a different genetic condition, and exome sequencing was performed in the familial trio. Whole-exome sequencing libraries were prepared using the SureSelect All Exon V8 kit (Agilent Technologies, Santa Clara, CA, USA) and sequenced with a mean depth of 100×. Reads were aligned and filtered and variants were called with the GATK software version 4.4.0.0 (Broad Institute, Cambridge, MA, USA). Variants in genes consistent with the patient’s overall clinical presentation, including genes associated with the renal phenotype and focal segmental glomerulosclerosis, were annotated and analyzed. The pathogenic *NOTCH3* variant NM_000435.2:c.3016C>T, p.(Arg1006Cys) was identified in a heterozygous state in the proband and in his father, and a genetic diagnosis of CADASIL was made in both. Additional heterozygous variants were detected in *CUBN* and *VARS2*, together with the previously identified *MKKS* variant, all genes associated with autosomal recessive conditions characterized by non-comparable clinical signs. The main molecular findings are summarized in Table 2.

### 2.3. Neuroimaging and CADASIL-Related Family History

After the finding of a pathogenic *NOTCH3* variant, the patient underwent brain MRI, including FLAIR, GRE, DWI, and T1- and T2-weighted sequences that allowed for the diagnosis of chronic vascular-type leukoencephalopathy with diffuse, partially confluent white matter signal abnormalities involving the centrum semiovale and corona radiata and mild ventricular enlargement. No ischemic lesions or intra/extra-axial hemorrhagic areas were detected; all of these findings were consistent with CADASIL-related small-vessel disease, without evidence of acute or lacunar infarcts.

**Figure 1:**
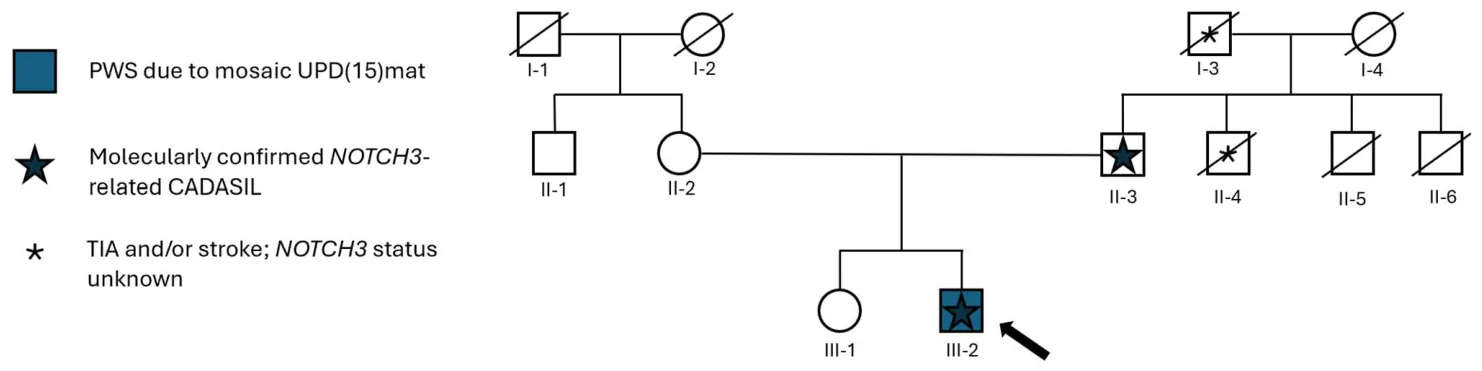
Three-generation pedigree of the proband’s family. Squares and circles represent males and females, respectively; diagonal lines indicate deceased individuals, and the arrow identifies the proband. Teal fill denotes PWS due to mosaic UPD(15)mat; stars indicate individuals with molecularly confirmed *NOTCH3*-related CADASIL; asterisks indicate relatives with a history of transient ischemic attacks and/or stroke whose status for the familial *NOTCH3* variant was unknown. Roman numerals indicate generations, and Arabic numerals identify individuals within each generation.

The family history was notable for cerebrovascular events on the paternal side (Figure 1). The patient’s father, who had experienced recurrent transient ischemic attacks, carried the same *NOTCH3* variant identified in the proband. A paternal uncle had also experienced transient ischemic attacks and died from a stroke at approximately 60 years of age; however, he had not undergone molecular testing for the familial *NOTCH3* variant. In addition, the paternal grandfather reportedly suffered a stroke in his early forties and died at 65 years of age. Although the proband had not experienced overt cerebrovascular events, the diagnosis of CADASIL was established by the pathogenic *NOTCH3* variant and further supported by the concordant MRI findings and the segregation of the variant with cerebrovascular disease in his father. The additional cerebrovascular events in the paternal lineage were consistent with the autosomal dominant family history, although no molecular diagnosis was available for the paternal uncle or grandfather. The proband was therefore considered to have a currently oligosymptomatic presentation of CADASIL.

## 3. Discussion

The patient we describe underwent a long follow-up for the clinical suspicion of Prader–Willi syndrome that was not confirmed by repeated molecular tests performed on peripheral blood during his first 25 years of life. Furthermore, during adulthood, in addition to the expected signs of PWS, he presented with a more complex phenotype including severe metabolic impairment, liver dysfunction, and focal segmental glomerulosclerosis. A family history of recurrent ischemic attacks and late-onset cognitive decline was also recorded.

Driven by the clinical suspicion of PWS, the more sensitive SNP-array and 15q11q13-specific MS-MLPA analyses were carried out on DNA derived not only from blood cells but also from buccal mucosal cells, and the diagnosis of mosaic UPD(15)mat was made, involving approximately 10% and 40% of cells in blood and buccal mucosa, respectively. In addition, prompted by the clinical suspicion of a comorbidity of two genetically independent conditions, exome sequencing was performed and revealed a familial pathogenic *NOTCH3* variant causative of CADASIL.

In more detail, our patient had a history suggestive of PWS since birth. Over time, he presented with low birth weight, severe neonatal hypotonia with poor sucking, early hyperphagia, severe obesity, short stature, hypogonadism-related features, and growth hormone deficiency. Nevertheless, repeated methylation-based analyses on peripheral blood cells failed to confirm the diagnosis of PWS for decades. The final diagnosis became possible only when the clinical hypothesis was maintained and testing was extended to a second tissue, where the mosaic level was higher than in blood. Importantly, SNP-array revealed a ~10% mosaic UPD(15)mat in peripheral blood that was not detectable by MS-MLPA of the 15q11q13 region on the same tissue, highlighting the limited sensitivity of methylation-based testing for low-level mosaicism.

This case is consistent with previous reports showing that mosaic UPD(15)mat may be missed by conventional diagnostic approaches, particularly when abnormal cells are under-represented in peripheral blood [5,6,7,8,9,10,11,12]. SNP-array is especially useful when mosaic UPD produces a copy-neutral loss of heterozygosity pattern, while MS-MLPA can provide complementary methylation and dosage information and may help estimate mosaicism. Accordingly, in selected patients with a convincing PWS phenotype despite normal blood-based methylation studies, we suggest the following stepwise approach: (i) re-evaluate the clinical phenotype and the first-line assay, including its sensitivity for low-level mosaicism; (ii) perform SNP-array analysis on peripheral-blood DNA, if not already undertaken, to assess copy-number changes and copy-neutral regions of homozygosity compatible with isodisomy; and (iii) if SNP-array suggests low-level mosaicism, or if blood studies remain non-diagnostic despite persistent clinical suspicion, analyze DNA from a second tissue, such as buccal epithelium, by MS-MLPA and, when feasible, SNP-array. Because SNP-array does not detect all PWS molecular mechanisms, it should complement rather than replace methylation analysis [1,4].

**Figure 2:**
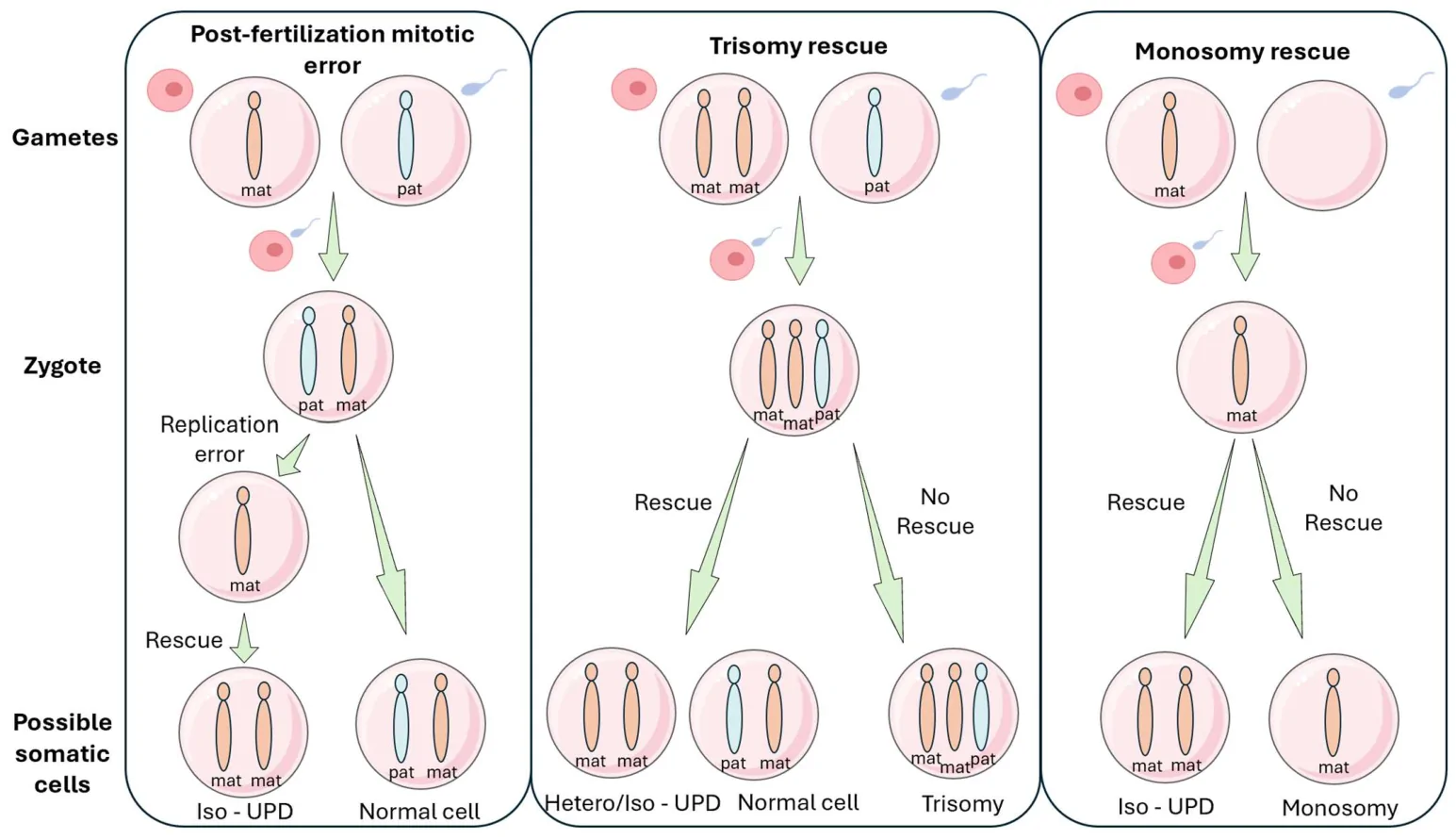
Overview of the biological mechanisms leading to uniparental hetero- and isodisomy. Figure adapted using Servier Medical Art (https://smart.servier.com, accessed on 22 June 2026), licensed under CC BY 4.0.

Mosaic maternal UPD(15) may arise through different post-zygotic or rescue mechanisms (Figure 2). In the classical trisomy rescue model, a conceptus initially trisomic for chromosome 15 loses one chromosome 15; if the paternal chromosome is lost, the remaining chromosome 15 pair is maternal, usually resulting in maternal heterodisomy or mixed heterodisomy/isodisomy. In contrast, the considerably rarer event of complete maternal isodisomy in a mosaic state is more suggestive of a post-fertilization mitotic error or monosomy rescue mechanism: after the loss of the paternal chromosome 15 in one embryonic cell, duplication of the remaining maternal chromosome can generate a clone with maternal isodisomy [isoUPD(15)], while the other cell line remains with a biparental chromosome complement. Based on this model, we can infer the presence of mosaic maternal isoUPD(15) in our patient, with different mosaic levels observed in blood and buccal cells [5,6,7,8,9,10,11,12].

To contextualize the present observation, we reviewed a total of nine published postnatal cases in which a PWS or PWS-like phenotype was associated with mosaic UPD(15)mat, either as isolated UPD mosaicism or as UPD(15)mat mosaicism with residual trisomy 15 mosaicism (Table 3).

A great variability of the clinical phenotype is observed, according to the expected variable degree of mosaic UPD(15) in individual patients.

The neurodevelopmental profile was relatively mild in our patient, compared with many classical PWS cases. Conversely, the endocrine-metabolic disease was severe, with childhood-onset hyperphagia, morbid obesity, severe insulin resistance, type 2 diabetes mellitus, hypertension, dyslipidemia, hyperuricemia, and progressive MASLD/MASH with fibrosis. This dissociation supports the concept that the relative abundance and tissue distribution of the mosaic cell line may influence organ-specific disease burden.

The proportions estimated in blood and buccal cells cannot be assumed to reflect the mosaic burden in inaccessible organs. A relatively higher proportion of UPD(15)mat cells in hypothalamic circuits involved in satiety and neuroendocrine regulation could contribute to hyperphagia, growth hormone deficiency, hypogonadism, and severe metabolic dysregulation, whereas a lower burden in cortical and other neurodevelopmentally relevant tissues might be compatible with the relatively preserved cognitive profile. Similarly, variation in the mosaic involvement of hepatic, adipose, or pancreatic tissues could theoretically modulate susceptibility to MASLD/MASH, insulin resistance, and diabetes. However, these interpretations remain speculative because these tissues were not tested, and the severe hepatic and metabolic phenotype may also be explained, at least in part, by the downstream effects of long-standing obesity and diabetes.

The identification of the *NOTCH3* NM_000435.2:c.3016C>T, p.(Arg1006Cys) variant provided a second diagnosis. This cysteine-altering variant is a well-established CADASIL-associated change and is recognized as a recurrent/founder variant in central Italy, namely, in the Marche region [16]. Its paternal inheritance, together with the paternal history of transient ischemic attacks and early cerebrovascular disease in the paternal lineage, strongly supports its clinical relevance. The brain MRI detection of a vascular-type white matter injury without acute ischemia or hemorrhage, consistent with a small-vessel disease, was in strong concordance with the molecular diagnosis of CADASIL. In the present patient, the CADASIL diagnosis is of particular clinical relevance, since it adds a further, independent vascular risk on top of that conferred by diabetes, obesity, and hypertension, allowing for more accurate surveillance and therapy.

A final consideration is warranted regarding the renal phenotype of focal segmental glomerulosclerosis and interstitial fibrosis. Renal involvement in CADASIL has been reported only in a limited number of patients. Reported clinical findings have included proteinuria, microscopic hematuria, and mild or variable renal dysfunction. From a histopathological perspective, reported findings include granular osmiophilic material and NOTCH3 extracellular-domain accumulation in renal arterioles, interlobular, and juxtaglomerular arteries, together with vascular smooth muscle cell loss, nephroangiosclerosis-like changes, and arteriolar stenosis [17,18,19,20].

Therefore, *NOTCH3*-related arteriopathy can also affect the kidney, as expected. However, focal segmental glomerulosclerosis has not been clearly described in CADASIL, and the present case cannot establish it as an additional manifestation of the disease. Experimental data provide indirect biological plausibility for a role of Notch3 signaling in glomerular injury but do not demonstrate that CADASIL-associated *NOTCH3* variants cause focal segmental glomerulosclerosis.

El Machhour and colleagues [21] reported that Notch3 is virtually absent in normal glomeruli but is expressed in podocytes of patients with focal segmental glomerulosclerosis and other glomerular diseases. In a murine model of rapidly progressive glomerulonephritis, podocyte Notch3 expression was induced several-fold in parallel with disease progression, driving podocyte phenotypic changes, inflammation, proteinuria, and renal functional decline, whereas Notch3-deficient mice were protected. Other studies support the role of Notch pathway reactivation in podocyte injury and renal fibrosis [22,23,24]. These experimental results support the hypothesis that Notch3 signaling can participate in glomerular injury pathways.

Alternative explanations have to be considered. In our patient, proteinuria and microalbuminuria were detected from 22 years of age, and renal biopsy at 28 years showed focal segmental glomerulosclerosis, global sclerosis in two glomeruli, focal fibrous thickening of Bowman’s capsule in two glomeruli, intimal hyalinosis of arterioles, and areas of tubular atrophy associated with interstitial fibrosis and mild lymphocytic inflammation. Direct immunofluorescence showed only trace IgM and C3 deposits. Overall, this pattern is compatible with chronic glomerular and tubulointerstitial injury with vascular/metabolic features, rather than with immune-complex glomerulonephritis. Furthermore, no data are available to support the hypothesis of a CADASIL-specific pattern of renal injury.

Secondary/adaptive FSGS can occur in the setting of severe obesity, diabetes mellitus, hypertension, reduced nephron reserve, or chronic kidney stress, and obesity-related glomerulopathy is a recognized secondary form of FSGS [25,26]. This patient had long-standing morbid obesity, severe insulin resistance, type 2 diabetes, hypertension, hyperuricemia, recurrent urinary tract infections, and bladder dysfunction. In addition, the presence of arteriolar hyalinosis is consistent with chronic vascular and metabolic injury, particularly in the context of diabetes and hypertension. Therefore, a multifactorial mechanism combining complex metabolic and hemodynamic stress with a possible, but unproven, contribution of *NOTCH3*-related renal microangiopathy appears to be the most plausible explanation for the renal injury.

Accordingly, this single observation should be regarded as hypothesis-generating only. Systematic renal assessment in larger CADASIL cohorts will be required to determine whether FSGS occurs more frequently than expected.

## 4. Conclusions

Mosaic maternal isoUPD(15) can cause a clinically meaningful PWS phenotype and may be missed when the abnormal cell line is under-represented in peripheral blood. Aligned with the most recent diagnostic guidelines [4], our case underscores the critical importance of clinical suspicion in the diagnostic process. In patients with clinical evidence of PWS and negative first-line testing, the analysis of a second tissue and integration of SNP-array with MS-MLPA can be decisive. Further highlighting the pivotal role of clinical genetics, a dual diagnosis of mosaic UPD(15)mat and CADASIL was driven by the extensive evaluation of the patient’s phenotype and of the family history as well. The final diagnosis allowed for proper surveillance and therapeutics for vascular risks.

Finally, the renal phenotype should be interpreted cautiously. Published human data support renal vascular involvement in CADASIL, whereas experimental studies implicate Notch3 signaling in glomerular injury; however, neither establishes a causal association between CADASIL and focal segmental glomerulosclerosis. In the present patient, the renal lesion is more plausibly explained by multifactorial secondary/adaptive injury, although an unproven contribution of *NOTCH3*-related renal microangiopathy cannot be excluded.

## Figures and Tables

**Table 1:** Main clinical and diagnostic timeline.

Age	Clinical or Diagnostic Event
Birth/neonatal period	Term birth, birth weight 2450 g, neonatal distress, hypotonia, poor sucking, incubator stay
1–7 years	Hyperphagia, progressive weight gain, delayed motor/language milestones
4 years	Surgery for unilateral cryptorchidism
12–17 years	Growth hormone therapy; pituitary hypoplasia documented on MRI
13.8 years	Impaired glucose tolerance with marked hyperinsulinemia
~20 years	Type 2 diabetes mellitus
Adulthood	MASLD/MASH with fibrosis, severe obesity, hypertension, dyslipidemia, hyperuricemia
22–28 years	Proteinuria/microalbuminuria; renal biopsy: FSGS, arteriolar hyalinosis, mild tubulointerstitial fibrosis
40 years	SNP-array/MS-MLPA on blood and buccal smears: mosaic UPD(15)mat
43 years	Exome sequencing: *NOTCH3* NM_000435.2:c.3016C>T, p.(Arg1006Cys)

**Table 2:** Summary of genetic investigations.

Test	Tissue/Sample	Result	Interpretation
Karyotype	Peripheral blood	46,XY	No chromosomal abnormality detected by conventional cytogenetics
BBS-related testing	Peripheral blood	Heterozygous *MKKS*/*BBS6* NM_170784.3:c.724G>T variant	Carrier/VUS-type finding; not sufficient for recessive BBS
Repeated PWS methylation analyses	Mainly peripheral blood	Repeatedly normal/uninformative	False-negative/non-diagnostic due to low-level mosaicism in blood
SNP-array	Peripheral blood	Mosaic maternal UPD(15), approximately 10%	Evidence of mosaic UPD(15)mat
SNP-array/MS-MLPA	Buccal cells	Mosaic UPD(15)mat/hypermethylation, approximately 40%	Confirmed PWS; buccal tissue more informative than blood
Exome sequencing	Peripheral blood, trio analysis	*NOTCH3* NM_000435.2:c.3016C>T, p.(Arg1006Cys), paternally inherited	Molecular and clinical diagnosis of CADASIL
Additional NGS findings	Peripheral blood	Heterozygous variants in *MKKS*, *CUBN* and *VARS2*	Carrier state for recessive conditions; not explanatory

**Table 3:** Published postnatal cases of PWS/PWS-like phenotypes associated with mosaic UPD(15)mat or UPD(15)mat with residual trisomy 15 mosaicism.

Reference	Sex/Age	Mosaic UPD(15) Finding	Main Reported Phenotype	Diagnostic/Interpretative Note
Milunsky et al., 1996 [5]	F/liveborn infant	Trisomy 15 mosaicism with maternal UPD(15) in lymphocytes	IUGR; multiple congenital anomalies reported at birth	Early evidence that suspected trisomy 15 mosaicism may require testing of more than one tissue.
Devriendt et al., 1997 [6]	F/3.3 years	Maternal heterodisomy 15 in blood; trisomy 15 cell line in fibroblasts; additional mosaic 47, XXX cell line	PWS phenotype	Direct evidence for trisomic rescue by reduction to disomy as a basis for PWS.
Olander et al., 2000 [7]	M/infancy	Maternal isodisomy 15 with mosaic trisomy 15	Hypotonia, PWS facial phenotype, growth/developmental delay, congenital heart defects	Represents UPD(15)mat with residual trisomy 15 mosaicism.
Horsthemke et al., 2003 [8]	F	UPD(15)mat cells in blood and skin	PWS phenotype; mild neonatal hypotonia without tube feeding, SGA, developmental delay, obesity and cognitive impairment	Somatic mosaicism confirmed by cloning and genotyping of skin fibroblasts.
Izumi et al., 2013, Patient 1 [9]	F/infant	Mosaic maternal isodisomy of the entire chromosome 15	PWS diagnosed in infancy; neonatal hypotonia and SGA reported in the comparative literature	SNP-array identified a normal cell line and supported a post-fertilization error mechanism.
Izumi et al., 2013, Patient 2 [9]	F/infant	Mosaic maternal mixed iso/heterodisomy 15 with a trisomic cell line	PWS diagnosed in infancy; neonatal hypotonia and SGA reported in the comparative literature	SNP-array detected low-level mosaicism and supported trisomy rescue.
Wang et al., 2013 [10]	F	Mosaic isochromosome 15q with maternal uniparental isodisomy (~30% cells)	Morbid obesity, temper tantrums/behavioral features, short stature and hypogonadism; no neonatal hypotonia or intellectual disability	Milder neurocognitive involvement
Zilina et al., 2014 [11]	M	Mosaic maternal UPD15 (~55–60% cells)	Neonatal hypotonia, developmental delay, overweight and cognitive impairment; no hypogonadism or short stature	Partial PWS phenotype due to the limited endocrinological involvement
Morandi et al., 2015 [12]	F/17 years	Mosaic maternal isoUPD15 (~50% cells)	Severe obesity, short stature and intellectual disability; no neonatal hypotonia or hypogonadism	Methylation-sensitive PCR was normal; SNP-array and quantitative methylation confirmed partial PWS due to mosaic UPD15.

## Data Availability

The data presented in this article are available from the corresponding author upon reasonable request, subject to privacy and ethical restrictions.

## Abbreviations

The following abbreviations are used in this manuscript:

BBS	Bardet–Biedl syndrome
BMI	Body mass index
C3	Complement component 3
CADASIL	Cerebral autosomal dominant arteriopathy with subcortical infarcts and leukoencephalopathy
DWI	Diffusion-weighted imaging
EGF	Epidermal growth factor
FLAIR	Fluid-attenuated inversion recovery
FSGS	Focal segmental glomerulosclerosis
GRE	Gradient echo
HbA1c	Glycated hemoglobin
HDL	High-density lipoprotein
HOMA-IR	Homeostatic model assessment of insulin resistance
IgM	Immunoglobulin M
MASH	Metabolic dysfunction-associated steatohepatitis
MASLD	Metabolic dysfunction-associated steatotic liver disease
MLPA	Multiplex ligation-dependent probe amplification
MRI	Magnetic resonance imaging
MS-MLPA	Methylation-specific multiplex ligation-dependent probe amplification
NAS	NAFLD activity score
NGS	Next-generation sequencing
PCR	Polymerase chain reaction
PWS	Prader–Willi syndrome
SGA	Small for gestational age
SNP	Single-nucleotide polymorphism
UPD	Uniparental disomy
VUS	Variant of uncertain significance
